# A case report of unilateral cerebral vasculitis in adults: keep in mind Lyme neuroborreliosis

**DOI:** 10.1186/s12879-023-08259-z

**Published:** 2023-05-04

**Authors:** Stanislas Riescher, Amélie Dos Santos, Raphaël Lecomte, Cédric Lenoble, Benoit Guillon

**Affiliations:** 1 Service de Médecine interne, Nantes University Hospital Center, Nantes cx 1, France; 2 Service de Neurologie, Nantes University Hospital Center, Nantes, France; 3 Service de Maladies infectieuses et Tropicales, Nantes University Hospital Center, Nantes, France

**Keywords:** Neuroborreliosis, Lyme, Vasculitis, Stroke, Case report

## Abstract

**Background:**

Lyme neuroborreliosis (LNB), due to infection of the nervous system by the spirochete *Borrelia burgdorferi*, occurs in 15% of Lyme disease cases. However, neurovascular involvement is uncommon, especially recurrent stroke related to cerebral vasculitis in the absence of CSF pleocytosis.

**Case presentation:**

We report the case of a 58-year-old man without any medical history who exhibited recurrent strokes in the same vascular territory (left internal carotid). Multiple biological screening, neuroimaging methods, and cardiovascular examinations failed to provide a diagnosis and treatment that could have prevented recurrences. Finally, *B. burgdorferi sensu lato* serology testing in blood and cerebrospinal fluid enabled diagnosis of LNB, in relation to a cerebral vasculitis. The patient experienced no further stroke after four weeks of doxycycline treatment.

**Conclusion:**

*B. burgdorferi* central nervous system infection must be considered in case of unexplained recurrent and/or multiple strokes, especially if cerebral vasculitis is suspected or demonstrated on neuroimaging.

## Background

Lyme neuroborreliosis (LNB) corresponds to affliction of the central and/or peripheral nervous system secondary to systemic infection by *Borrelia burgdorferi*, which is a spirochete transmitted by tick bites (genus *Ixodes*). Neurological complications occur in up to 15% of untreated Lyme disease cases, mostly involving lymphocytic meningitis, meningoencephalitis, and cranial or radiculoneuritis [1]. Neurovascular manifestations are far less common, usually associated with CSF pleocytosis, and are related to cerebral vasculitis leading to stroke [2–7], sinus thrombosis, subarachnoid [8], or intracerebral hemorrhage [9]. Cerebral vasculitis is reported in 0.3–1% [10, 11] of all LNB. Cases of unilateral or focal vasculitis have only rarely been described, almost exclusively in children [4–6].

Here, we report the case of a 58-year-old man with recurrent strokes in the same vascular territory without CSF pleocytosis, reflecting an unusual first manifestation of LNB.

## Case presentation

**Fig. 1:**
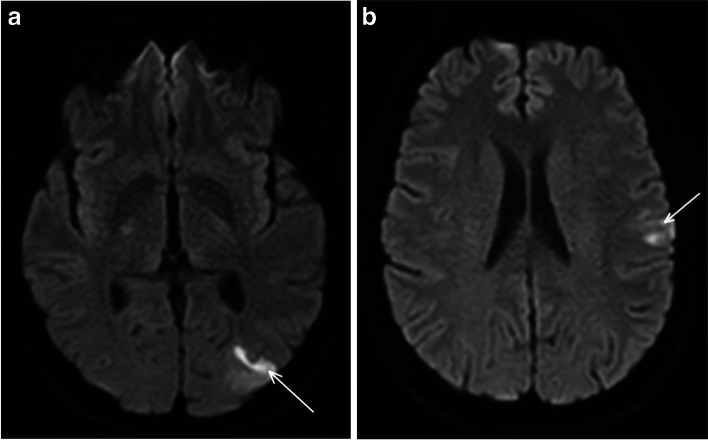
Concomitant acute ischemic lesions in the left MCA territory, in the parieto-occipital (**a**) and the frontal lobe (**b**); axial diffusion-weighted imaging DWI MRI

A 58-year-old man, without any medical history or cardiovascular risk factors, was admitted to the emergency department for sudden right brachiofacial weakness. He recovered partially, but facial palsy persisted. Brain DWI-MRI revealed two cortical infarcts in the left middle cerebral artery (MCA) territory, in the parieto-occipital and the frontal lobe (Fig. 1).

He was then referred to our Stroke Unit. The neurological and general examination was normal, except for moderate hypertension (SBP between 140 and 150 mmHg). Cervical and intracranial CT angiography did not show any abnormalities such as stenosis, occlusion, or dilatation. Forty-eight hours of telemetry and transthoracic echocardiography (TTE) did not detect any abnormalities. Transesophageal echocardiography revealed a significant patent foramen ovale (PFO) without an atrial septal aneurysm. Biological testing including homocysteine, glycemia, lipid profile, anti-cardiolipin antibodies, and lupus anticoagulant toxic screening was normal.

At discharge, he was treated with aspirin (160 mg), atorvastatin (80 mg), and perindopril at 2 mg daily. The diagnostic conclusion was a cryptogenic stroke, and a multidisciplinary meeting was held to discuss the relationship between PFO and stroke for possible closure. The PFO was considered to be “high-risk” as there was a large right-to-left shunt with more than 30 microbubbles in the left atrium within the three cardiac cycles of seeing opacification in the right atrium. The RoPE (Risk of Paradoxical Embolism) score was 7, indicating that the PFO was causally related to the cryptogenic stroke, with an attributable fraction of 72% [12].

**Fig. 2:**
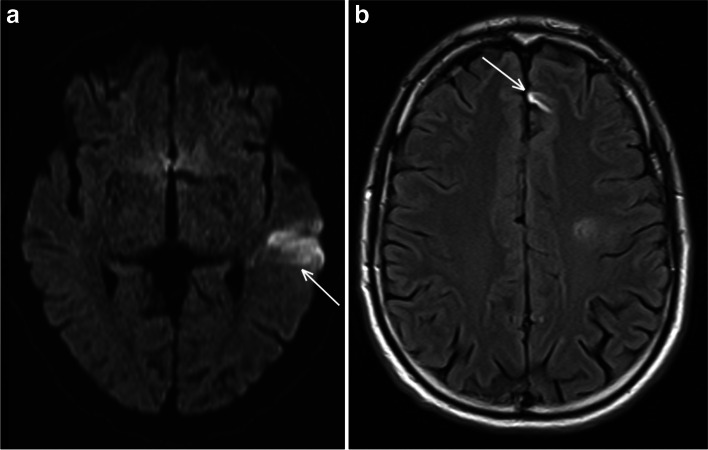
Occurrence of two new lesions compared to in the previous MRI. A left temporal lobe hypersignal on axial DWI MRI related to a recent ischemic lesion in the left MCA territory (**a**) and an older one in the ACA territory, only visible on axial FLAIR sequences (**b**)

Two months later, when the patient was back to work, he exhibited sudden aphasia. MRI revealed a new and recent ischemic lesion in the left MCA territory visible on DWI, and another older lesion in the anterior cerebral artery (ACA) territory, which had been absent on the previous imaging (Fig. 2). There was no wall thickening nor wall enhancement on high-resolution contrast-enhanced MRI.

Venous ultrasound examination of the lower limb was normal, and a body CT scan excluded an inflammatory or neoplastic lesion or large vessel vasculitis. A more extensive thrombophilia screening, including antithrombin III, Factor II and V Leiden mutations, C and S proteins, antiphospholipid antibodies, and inflammatory markers (CRP), was unremarkable.

In line with the advice from cardiologists, aspirin was replaced with an oral anticoagulant (5 mg apixaban twice daily), to close the PFO and to insert a cardiac monitor.

The PFO closure was performed 8 months after the first stroke, after ensuring that there was no paroxysmal rhythm disorder on the cardiac monitor during the previous 6 months.

During the following months, the patient complained of new symptoms consisting of paresthesia in the right hand and foot, posterior headache, asthenia, psychomotor retardation, and unsteadiness, and thirteen months after the first symptoms he presented right leg paresis. A brain MRI showed a new recent ischemic lesion in the left cingulate cortex on DWI in the left ACA territory as well as other new lesions in the left MCA territory (in the frontal lobe) compared with the previous MRI 12 months earlier. There was still no vessel stenosis or occlusion and no wall enhancement.

**Fig. 3:**
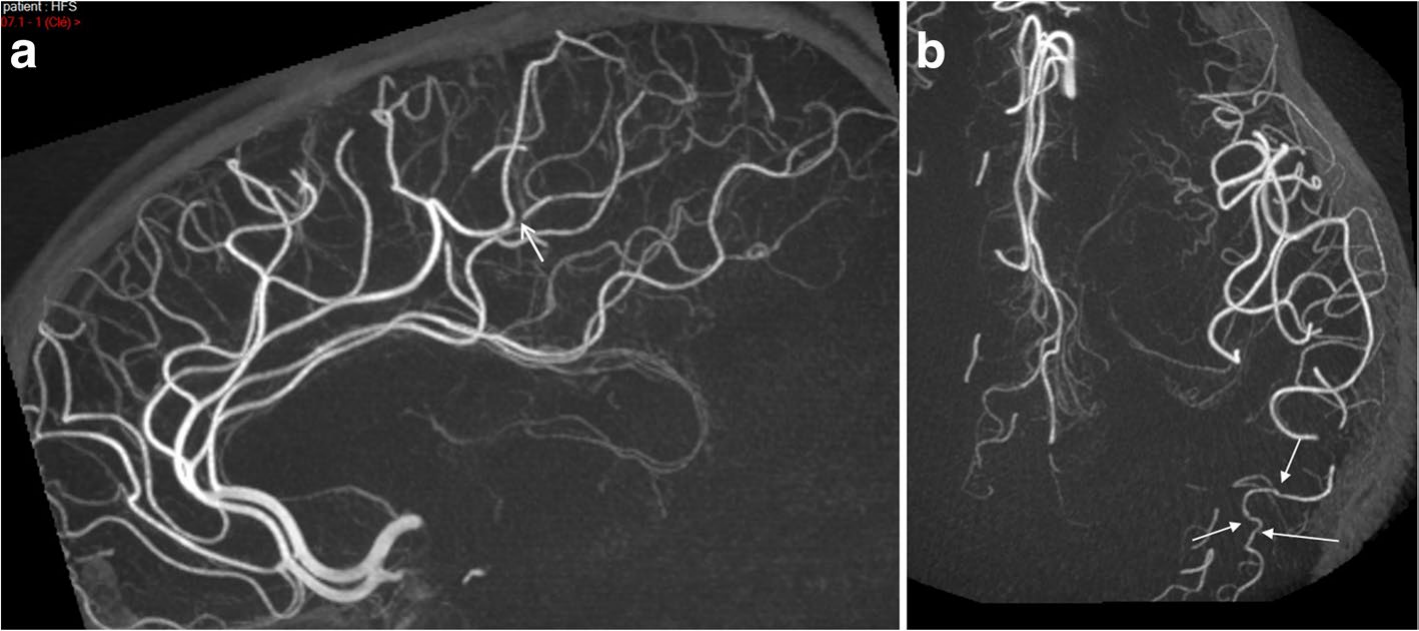
Digital subtraction angiography. **a** Severe stenosis of a distal branch of the left anterior cerebral artery (arrow); sagittal view of both anterior cerebral arteries. **b** Mild distal irregularities in the left posterior temporal artery (arrows), a branch of the middle cerebral artery; axial view

The biological screening was resumed (anti-phosphatidylethanolamine and anti-annexin V; JAK2-CALR-MPL mutation; antinuclear factor; rheumatoid factor; ANCA; cryoglobulin; multiple serologies including HIV, B, and C hepatitis; syphilis; QuantiFERON-TB®-Gold; alpha-galactosidase activity; and paroxysmal nocturnal hemoglobinuria clonal component) but remained negative. Ophthalmic examination by fluorescein angiography was normal. On lumbar puncture, there was no meningitis or protein increase, no bacteria visualized or virus (PCR), nor intrathecal synthesis. New TTE and cervical ultrasound remained normal. Electroneuromyography showed an isolated deceleration in the left ulnar nerve. Digital subtraction angiography (DSA) showed narrowing in a distal branch of the left ACA and in the posterior temporal artery consistent with intracranial vasculitis (Fig. 3). Anticoagulation was replaced by dual antiplatelet therapy (aspirin 75 mg + clopidogrel 75 mg).

Finally, blood *B. burgdorferi sensu lato* serology testing was highly positive, with IgG 118 U/mL on ELISA, confirmed by Western blot. The patient was then hospitalized one month later for a new CSF examination. The serology was still positive on ELISA but doubtful on immunoblot IgG (only one band, IgG VIsE-Mix positive against two required for confirmation). There was still no sign of meningitis on CSF analysis, but the serology was highly positive, with IgG 18 IU/mL [high if > 9] and intrathecal synthesis of specific antibody [index > 2]. Doxycycline 200 mg twice daily was then initiated for four weeks, with good tolerability.

In the following weeks, the patient reported dramatic improvement, with disappearance of his asthenia, dizziness, and right arm paresthesia. Finally, upon re-interviewing of the patient, he reported a tick bite two years prior to his first stroke, with a skin lesion compatible with erythema migrans, but he never suffered from arthritis or pain, nor did he have clinical signs of cranial or radiculoneuritis.

Atorvastatin and clopidogrel were stopped, maintaining only aspirin.

**Fig. 4:**
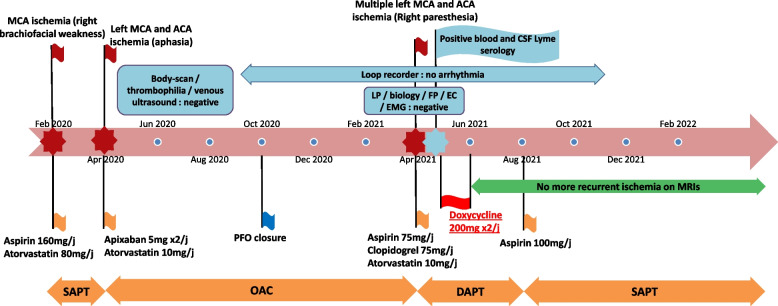
Case timeline. *ACA: anterior cerebral artery – MCA: middle cerebral artery – SAPT: single antiplatelet therapy – DAPT: dual antiplatelet therapy – OAC: oral anticoagulation – LP: lumbar puncture – FP: fundus photography – EC: echocardiography – EMG: electromyogram – PFO: patent foramen ovale*

On follow-up, there was no new ischemic lesion on MRI performed 5 months after the end of treatment, and he experienced no further cerebrovascular events (last visit 18 months post antibiotic therapy). At the last visit, the patient reported that he had recovered without any sequelae and had resumed all his previous activities including sports (Fig. 4).

## Discussion and conclusions

This case highlights diagnostic difficulties for patients with LNB cerebrovascular manifestations. Our patient stood out because he had recurrent ischemic strokes for more than one year (at least four episodes), without other associated symptoms, involving the ICA territory only, and without CSF pleocytosis, which is thought to be highly suggestive of LNB.

Our diagnosis of LNB unilateral vasculitis was based on (1) possible neuroborreliosis according to European guidelines, with positive *B. burgdorferi’s* serology, intrathecal synthesis of specific antibodies, and an extensive work-up ruling out other diagnoses [13]; (2) a compatible history of a tick bite two years before the first stroke associated with a possible erythema chronic migrans; (3) recurrent strokes in a single arterial territory (left ICA), without cardiologic, biological, or vascular underlying lesions, except distal mild irregularities suggestive of distal intracranial vasculitis on DSA, and (4) dramatic improvement after specific treatment of LNB, with no further recurrent strokes during follow-up. Blanc et al. proposed five pragmatic criteria to confirm neuroborreliosis, comprising no past history of neuroborreliosis, positive CSF ELISA serology, positive anti-Borrelia antibody index, favorable outcome after specific antibiotic treatment, and no differential diagnosis. All criteria were validated in our patient [14].

However, we acknowledge that the usual picture of LNB is lacking, such as CSF pleocytosis or clear ICA damage (to explain the one-sided infarcts), as well as a lack of usual features of vasculitis, such as wall contrast enhancement on MRI or major irregularities on CT angiogram or DSA, making the diagnosis more difficult. A much more extensive screening was performed in order to understand the mechanism of the strokes, initially attributed to PFO. This diagnosis was nevertheless uncertain, and a cardioembolic mechanism was doubtful because of these multiple recurrences within a single carotid artery territory and irrespective of the antithrombotics regimen. Finally, we can explain this rare phenomenon by unilateral cerebral vasculitis, even though a focal lesion of the left carotid arterial wall leading to thromboembolization in the downstream flow, not visible in imaging studies, cannot be excluded.

Stroke is a rare manifestation of LNB, accounting for < 1% of cases [15], and when present, related to cerebral vasculitis [10, 11]. Cases of focal or unilateral LNB vasculitis have only rarely been described, mainly in children [4–6]. Imaging studies should reveal multiple short stenoses at branching sites of the intracranial artery [4], single regular segmental stenosis [5], or wall contrast enhancement of the basilar artery wall [6].

LNB vasculitis can affect either large/medium-sized arteries causing dilatation, stenosis, and/or wall contrast enhancement, or small blood vessels, showing no abnormalities on medical imaging [10]. Nevertheless, MRI appears to be the most sensitive imaging method, revealing parenchymal damage and meningeal or wall vessel enhancement. In a systematic review including 63 patients [10], a history of tick bites was present in only 58.8% of cases, and extra-neurological signs were noted in < 9%, whereas a history of headache and/or of radiculitis or cranial neuritis were noted in two-thirds of cases. There was lymphocytic pleocytosis on CSF examination (median of 77 cells/ml) in more than 95% of patients. Furthermore, vasculitis was predominant in large-sized vessels (63.6%), in the posterior (37.8%) and the anterior and posterior (37.8%) circulation.

Treatment is based on appropriate antibiotics, most often ceftriaxone, but sometimes doxycycline is used [16]. More than 75% of patients had a complete response to treatment. However, some cases may require additional immunosuppressive therapy, such as cyclophosphamide [10].

The pathophysiology of LNB vasculitis shares similarities with neurosyphilis, especially in the meningovascular form with “endarteritis obliterans”. In the main hypothesis, *B. burgdorferi* induce inflammation and invasion of the arterial wall, and after breaking through the blood-brain barrier, spread into the CNS to form scattered perivascular mononuclear cell infiltrates in the cerebral cortex [17, 18]. Vasculitis can develop by activation of endothelial cells and release of inflammation mediators. Lesions secondary to an immune response with immune complexes is another hypothesis [17].

In conclusion, *B. burgdorferi*-induced cerebral vasculitis is thought to be a very rare manifestation of LNB. But it must nonetheless be considered in the differential diagnosis of unexplained recurrent and/or multiple strokes. This case report reminds clinicians that it can occur without a history of a clear tick bite or erythema migrans, and cranial or peripheral neuritis and that the CSF examination can be normal. Serology for *B. burgdorferi* in blood and CSF should be considered once first-line etiologies have been ruled out, particularly because effective treatment is available. However, physicians should be aware of the large number of false positives on serologies, especially ELISA, in areas with low disease prevalence. ELISA should be systematically confirmed by Western blot to improve the specificity and CSF examination performed with intrathecal synthesis research. In addition, serologies but also intrathecal synthesis do not provide information regarding the active character of neuroborreliosis and must, therefore, be interpreted according to the context [13].

## Data Availability

The data that support the findings of this study are available from the corresponding author.

## Abbreviations

LNB: Lyme neuroborreliosis
MRI: Magnetic resonance imaging
DWI: Diffusion-weighted imaging
MCA: Middle cerebral artery
SBP: Systolic blood pressure
TTE: Transthoracic echocardiography
CT: Computed tomography
PFO: Patent foramen ovale
ACA: Anterior cerebral artery
DSA: Digital subtraction angiography
CSF: Cerebrospinal fluid
ICA: Internal carotid artery

## References

[1] Koedel U, Fingerle V, Pfister HW (2015). Lyme neuroborreliosis-epidemiology, diagnosis and management. Nat Rev Neurol.

[2] Zajkowska J, Garkowski A, Moniuszko A (2015). Vasculitis and stroke due to Lyme neuroborreliosis - a review. Infect Dis.

[3] Topakian R, Stieglbauer K, Nussbaumer K, Aichner FT (2008). Cerebral vasculitis and stroke in Lyme neuroborreliosis. Two case reports and review of current knowledge. Cerebrovasc Dis.

[4] Kohns M, Karenfort M, Schaper J (2013). Transient ischaemic attack in a 5-Year-old girl due to focal vasculitis in Neuroborreliosis. Cerebrovasc Dis.

[5] Cox MG, Wolfs TF, Lo TH (2005). Neuroborreliosis causing focal cerebral arteriopathy in a child. Neuropediatrics.

[6] Lebas A, Toulgoat F, Saliou G (2012). Stroke due to lyme neuroborreliosis: changes in vessel wall contrast enhancement. J Neuroimaging.

[7] Moreno Legast G, Schnider A, Nicastro N. Ischemic stroke: Do not forget Lyme Neuroborreliosis. Case Rep Neurol Med. 2018:1720725. 10.1155/2018/1720725 PMC5899873 29805824

[8] Chehrenama M, Zagardo MT, Koski CL (1997). Subarachnoid hemorrhage in a patient with Lyme disease. Neurology.

[9] Seijo Martínez M, Grandes Ibáñez J, Sánchez Herrero J (2001). Spontaneous brain hemorrhage associated with Lyme neuroborreliosis. Neurologia.

[10] Garkowski A, Zajkowska J, Zajkowska A (2017). Cerebrovascular manifestations of Lyme Neuroborreliosis-A systematic review of published cases. Front Neurol..

[11] Back T, Grünig S, Winter Y (2013). Neuroborreliosis-associated cerebral vasculitis: long-term outcome and health-related quality of life. J Neurol.

[12] Kent DM, Ruthazer R, Weimar C (2013). An index to identify stroke-related vs incidental patent foramen ovale in cryptogenic stroke. Neurology.

[13] Mygland Ã, Ljøstad U, Fingerle V (2010). EFNS guidelines on the diagnosis and management of European Lyme neuroborreliosis. Eur J Neurol.

[14] Blanc F, Jaulhac B, Fleury M (2007). Relevance of the antibody index to diagnose Lyme neuroborreliosis among seropositive patients. Neurology.

[15] Oschmann P, Dorndorf W, Hornig C (1998). Stages and syndromes of neuroborreliosis. J Neurol.

[16] Bremell D, Dotevall L (2014). Oral doxycycline for Lyme neuroborreliosis with symptoms of encephalitis, myelitis, vasculitis or intracranial hypertension. Eur J Neurol.

[17] Oksi J, Kalimo H, Marttila RJ (1996). Inflammatory brain changes in Lyme borreliosis. A report on three patients and review of literature. Brain.

[18] Meurers B, Kohlhepp W, Gold R (1990). Histopathological findings in the central and peripheral nervous systems in neuroborreliosis. A report of three cases. J Neurol.

